# Disruption of diapause induction by TALEN-based gene mutagenesis in relation to a unique neuropeptide signaling pathway in *Bombyx*

**DOI:** 10.1038/srep15566

**Published:** 2015-10-26

**Authors:** Kunihiro Shiomi, Yoko Takasu, Masayo Kunii, Ryoma Tsuchiya, Moeka Mukaida, Masakazu Kobayashi, Hideki Sezutsu, Masatoshi Ichida Takahama, Akira Mizoguchi

**Affiliations:** 1 Faculty of Textile Science and Technology, Shinshu University, Ueda 386-8567, Japan; 2 National Institute of Agrobiological Sciences (NIAS), Tsukuba 305-8634, Japan; 3 Center for Bioresource Field Science (CBFS), Kyoto Institute of Technology, Kyoto 606-8585, Japan; 4 Graduate School of Science, Nagoya University, Nagoya 464-8602, Japan

## Abstract

The insect neuropeptide family FXPRLa, which carries the Phe-Xaa-Pro-Arg-Leu-NH_2_ sequence at the C-terminus, is involved in many physiological processes. Although ligand–receptor interactions in FXPRLa signaling have been examined using *in vitro* assays, the correlation between these interactions and *in vivo* physiological function is unclear. Diapause in the silkworm, *Bombyx mori*, is thought to be elicited by diapause hormone (DH, an FXPRLa) signaling, which consists of interactions between DH and DH receptor (DHR). Here, we performed transcription activator-like effector nuclease (TALEN)-based mutagenesis of the *Bombyx DH-PBAN* and *DHR* genes and isolated the null mutants of these genes in a bivoltine strain. All mutant silkworms were fully viable and showed no abnormalities in the developmental timing of ecdysis or metamorphosis. However, female adults oviposited non-diapause eggs despite diapause-inducing temperature and photoperiod conditions. Therefore, we conclude that DH signaling is essential for diapause induction and consists of highly sensitive and specific interactions between DH and DHR selected during ligand–receptor coevolution in *Bombyx mori*.

Neuropeptide signaling is functionally diverse as a result of specific peptide ligands that preferentially activate particular receptor subtypes to perform physiological and developmental functions in animals, a process that developed through ligand–receptor coevolution^1^,^2^. Insect FXPRLa-family neuropeptides, which carry the Phe-Xaa-Pro-Arg-Leu-NH_2_ sequence at the C-terminus, play a role in many physiological processes, including diapause induction, pheromone biosynthesis, cuticular tanning, myostimulation, pupariation behavior, and termination of pupal diapause^3^. These FXPRLa neuropeptides are evolutionarily related to the vertebrate peptide neuromedin U (NMU), and G-protein coupled receptors (GPCRs) in the NMU receptor clade are activated by FXPRLa peptides^4^. The sensitivity and specificity of response of NMU receptors to individual FXPRLa peptides has been studied in detail for some insect species using *in vitro* assays^2^,^4^,^5^,^6^,^7^,^8^. The results suggest that the ligand–receptor interactions in FXPRLa signaling have two major characteristics—high specificity and pleiotropism, indicating that certain peptides activate their respective authentic receptor with high specificity and that closely related clusters of specific peptides activate related groups of receptors^2^. However, the relationship between these ligand–receptor interactions and *in vivo* physiological functions remains unclear.

In the silkworm (*Bombyx mori*) genome, FXPRLa neuropeptides are encoded by two genes: diapause hormone (DH)-pheromone biosynthesis activating neuropeptide (PBAN) *DH-PBAN* and *capa* (Supplementary Table S1)^9^. *DH-PBAN* encodes a polypeptide precursor consisting of five FXPRLa neuropeptides: DH, PBAN, and α-, β-, and γ-SGNPs (Subesophageal ganglion neuropeptides) (Fig. 1A)^10^. The *capa* gene encodes a polypeptide precursor consisting of three neuropeptides that contain an FXPRLa, CAPA-PK. The GPCRs related to the NMU receptor activated by these peptides are clustered in the phylogeny^11^. DH is well known as a neuropeptide hormone responsible for induction of embryonic diapause in *Bombyx*^12^. DH functions by acting on a GPCR, DH receptor (DHR; Fig. 1B) in the developing ovaries during pupal–adult development in females^13^. Previous studies showed that DH is a selective and sensitive ligand for DHR and is distinguished from other neuropeptides encoded by *DH-PBAN*^8^,^13^. Therefore, it was thought that diapause induction elicited by DH signaling consisted of interactions between DH and DHR. However, the relationship between DH–DHR interactions and diapause induction has not been investigated *in vivo*.

*Bombyx* embryonic diapause is a unique process of seasonal polyphenism that is induced transgenerationally as a maternal effect in the bivoltine strain. Progeny diapause is determined by the mother’s experience during embryonic development. When eggs are incubated at 25 °C under continuous darkness (25DD), the resultant female moths are able to lay diapause eggs. In contrast, incubation at 15 °C under continuous darkness (15DD) causes the resultant moths to lay non-diapause eggs. If eggs are incubated at 20 °C under continuous illumination (20LL) or darkness (20DD), the resultant moths produce diapause or non-diapause eggs, respectively^14^. Thus, progeny diapause is determined by environmental factors such as photoperiod and temperature during maternal embryogenesis. Recently, we showed that the embryonic *Bombyx* TRPA1 ortholog (*BmTrpA1*) acts as a thermosensitive channel that is activated at temperatures above ~21 °C and affects diapause induction through DH release during pupal–adult development^15^. Thus, some of the molecular mechanisms in the pathway leading from reception of environmental signals to expression of the diapause phenotype have been revealed.

Recently, genome editing strategies including transcription activator-like effector nucleases (TALENs) have advanced the efficiency of targeted gene mutagenesis in a wide variety of organisms including *Bombyx mori*^16^,^17^. In the TALEN strategy, errors in the nonhomologous end joining (NHEJ) repair of the targeted double-strand breaks result in the mutations usually consisting of small deletions and/or insertions, which cause frameshifts and truncations of open reading frames to disrupt the gene functions^17^. Thus, application of TALEN mutagenesis is suitable for analysis of *in vivo* physiological functions of *Bombyx* genes involved in diapause induction.

Here, we performed TALEN-based mutagenesis of *Bombyx DH-PBAN* and *DHR* to isolate the null mutants of those genes in a bivoltine strain. All mutant silkworms were fully viable and showed no abnormalities in the developmental timing of ecdysis and metamorphosis. However, female adults oviposited non-diapause eggs despite diapause-inducing temperature and photoperiod conditions. Therefore, we concluded that DH signaling is essential for diapause induction, which is independently accomplished by highly sensitive and specific interactions between DH and DHR selected through ligand–receptor coevolution in *Bombyx mori*.

## Results

### Construction of TALEN-based mutants of *DH-PBAN* and *DHR*

**Figure 1:**
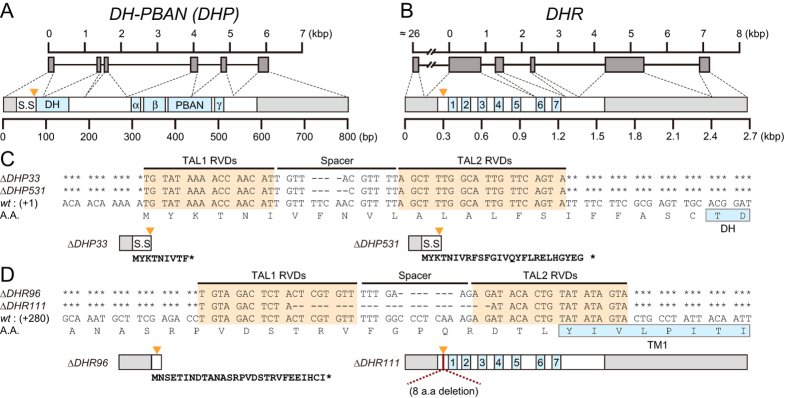
Construction of TALEN-based mutants of the *DH-PBAN* and *DHR* genes. Schematic representations of the genes (top) and cDNA structures (bottom) of the *DH-PBAN* (**A**) and *DHR* (**B**) mutants. Shaded boxes and lines represent exons and introns, respectively. The cDNA of *DH-PBAN* encodes a signal sequence (S.S), DH, PBAN, and α-, β-, and γ-SGNPs, which carry the 5′ and 3′ untranslated regions (light-grey boxes on left and right sides, respectively). The cDNA of *DHR* consists of seven transmembrane domains (1–7), which carry the 5′ and 3′ untranslated regions (light-grey boxes on left and right sides, respectively). The FXPRLa and transmembrane domains are indicated using blue boxes. Orange triangles represent the TALEN binding sites. The sizes of exons and introns (in bp) are indicated by scales in each map. (**C**,**D**) The sequences of the TALEN target sites are indicated by orange boxes. Partial coding sequences corresponding to the DH N-terminus and transmembrane domain 1 of DHR are indicated using blue boxes. The deleted bases in spacer regions of each mutant are indicated by hyphens, and identical bases are indicated by asterisks. The Δ*DHP33*, Δ*DHP531*, and Δ*DHR96* are truncated proteins, which encode 9, 26, and 28 amino acids, respectively. Eight amino acids were missing from *DHR111*, which led to the production of a defective truncated protein in the extracellular domain at the N-terminus of DHR.

For mutagenesis of *DH-PBAN*, we selected a TALEN target in the sequences of the first exon that encoded DH (Fig. 1A,C). We isolated the two homozygous mutants containing 4- or 5-base deletions and designated the mutants as Δ*DHP33* and Δ*DHP531*, respectively (Fig. 1C). These were considered null mutants, which could not translate the DH-PBAN preprohormone by frame-shift of *DH-PBAN* cDNA. We obtained two mutants of *DHR*, designated Δ*DHR96* and Δ*DHR111* (Fig. 1D), which carried a 7- or 24-base deletion, respectively, of the sequences in the anterior region encoding the transmembrane domain 1 (TM1) of the second exon (Fig. 1B). The Δ*DHR96* mutant translated the truncated protein containing a partial DHR signal sequence and was considered the null mutant. The Δ*DHR111* mutant was thought to cause an in-frame mutation; *DHR111* was missing eight amino acids, which led to the production of truncated protein defective in the extracellular domain at the N-terminus of DHR. Thus, we isolated four mutants related to DH signaling.

### DH and glycogen contents in Δ*DHP* and Δ*DHR* mutants

**Figure 2:**
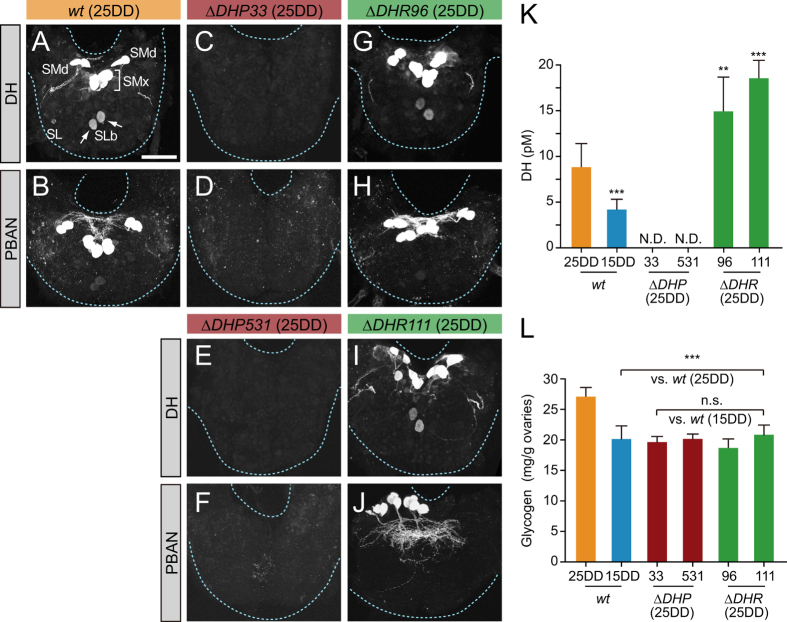
DH and glycogen contents in Δ*DHP* and Δ*DHR* mutants. (**A**–**J**) Representative images of immunohistochemical staining of pupal subesophageal ganglion (SG) using anti-DH[N] (**A**,**C**,**E**,**G**,**I**) and anti-PBAN[N] (**B**,**D**,**F**,**H**,**J**). The pupal SG was dissected 4 d after pupation, and immunostaining was performed in the *wt* (25DD) (**A**,**B**), Δ*DHP33* (25DD) (**C**,**D**), Δ*DHP531* (25DD) (**E**,**F**), Δ*DHP96* (25DD) (**G**,**H**), and Δ*DHR111* (25DD) (**I**,**J**). The DHPC somata (SMd, SMx, SLb, and SL) were detected in *wt* and Δ*DHR*. (**K**) The DH titer in the hemolymph was measured 4 d after pupation, by using TR-FIA. Each bar represents the mean ± SD of 8 samples. N.D., not detected; **P < 0.01; ***P < 0.001 vs. *wt* (25DD). (**L**) Glycogen content was measured in ovaries just after eclosion. Each bar represents the mean ± SD of 8 pupae. n.s., not significant vs. *wt* (15DD); ***P < 0.001 vs. *wt* (25DD). (Scale bar, 100 μm).

To confirm the null mutagenesis of *DH-PBAN*, we first investigated the immunoreactivity of DH and PBAN in pupal subesophageal ganglion (SG). Previous reports showed that *DH-PBAN* is exclusively expressed in seven pairs of neurosecretory cells (DH-PBAN-producing neurosecretory cells; DHPCs) located within the SG^18^,^19^. In *wt* (25DD) pupal SG, namely *wt* pupal SG that incubated at 25 °C under continuous darkness during embryogenesis, immunoreactive signals to anti-DH[N] antibody were detected in DHPC somata, including the SMd, SMx, and SLb neuromeres along the ventral midline and in SL cells (Fig. 2A), similar to previously reported results^20^. Likewise, immunoreactive signals were detected in DHPCs by using the anti-PBAN[N] antibody, although no SLb and SL cells were observed (Fig. 2B). Fluorescence signals disappeared in Δ*DHP33* and Δ*DHP531*, in which both anti-DH[N] and anti-PBAN[N] antibodies were used (Fig. 2C–F), suggesting that neither DH nor PBAN neuropeptides were produced in these mutants. In addition, pupal SG in Δ*DHR96* and Δ*DHR111* had immunoreactivity (Fig. 2G–J).

To further accurately determine the levels of circulating DH in the hemolymph, we developed a new, sensitive time-resolved fluoroimmunoassay (TR-FIA). By using synthetic DH as a standard, we found the detection limit for a 150-μL hemolymph sample from one or two animals to be ≈0.57 pM (≈85.8 amol). We measured DH levels in the hemolymph of pupa at 4 days after pupation (Fig. 2K). In *wt* (25DD) pupa, DH titer was 8.85 ± 2.71 pM, which was two-fold higher than in *wt* (15DD), suggesting the active release of DH in *wt* (25DD). DH was undetectable in the Δ*DHP33* and Δ*DHP531* lines despite rearing under 25DD conditions. Furthermore, DH levels in *DHR* mutants were two-fold higher than those in *wt* (25DD). These results suggest changes in DH titer in diapause of *wt*; in addition, DH levels were affected by the disruption of DH and DHR, indicating that DH signaling itself regulates the hemolymph DH levels.

*Bombyx* DH stimulates transcription of the trehalase gene in ovaries, thereby increasing trehalase activity, which facilitates greater accumulation of glycogen in eggs—a prerequisite for diapause initiation^21^,^22^. The glycogen content in ovaries of *wt* (25DD) pupa was high compared with that in *wt* (15DD) (Fig. 2L). The four 25DD Δ*DHP* and Δ*DHR* mutants had similar glycogen content to *wt* (15DD), but glycogen differed significantly from that in the *wt* (25DD). These results suggested that DH signaling affects glycogen accumulation in ovaries during the preparative phase of diapause.

### Phenotypic analyses of Δ*DHP* and Δ*DHR* mutants

**Figure 3:**
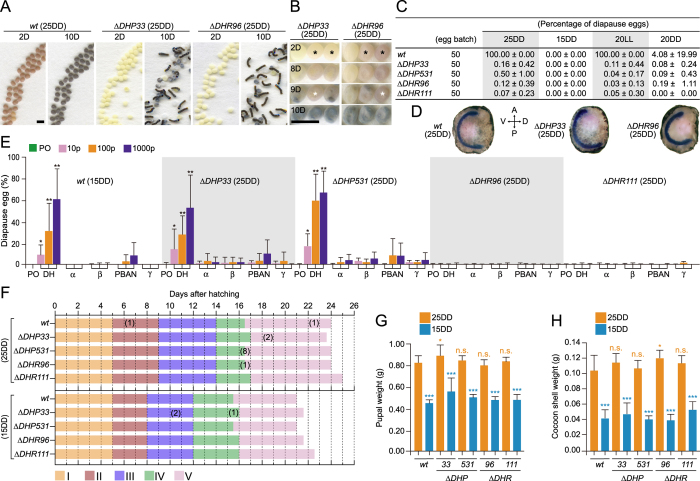
Phenotypic analyses of Δ*DHP* and Δ*DHR* mutants. (**A**) Representative images of eggs in *wt* (25DD), Δ*DHP33* (25DD), and Δ*DHP96* (25DD), 2 d or 10 d after oviposition. (**B**) Pigmented non-diapause eggs were observed in mutants. At 2 d after oviposition, pigmented eggs were observed (black asterisk); these eggs had delayed development (white asterisk). (**C**) Percentages of diapause eggs are shown for *wt* and four mutants incubated under various environmental conditions. The percentages of diapause eggs were estimated by counting the numbers of eggs in diapause and those not in diapause after non-diapause eggs hatched in 50 egg batches. (**D**) Morphological observation using thionin staining of egg. Embryos were collected 10 d after oviposition, and thionin staining was performed. We observed arrested development at diapause stage in early embryonic development in *wt* and in Δ*DHP33* (25DD) and Δ*DHR96* (25DD). (**E**) Rescue experiments by injection of synthetic FXPRLa peptides (DH, α-, β-, and γ-SGNPs, and PBAN) into female pupa of mutants. The peanut oil (PO) or each peptide (10, 100, and 1000 pmol) were injected into *wt* (15DD), Δ*DHP33* (25DD), Δ*DHP531* (25DD), Δ*DHR96* (25DD), and Δ*DHR111* (25DD), and the diapause eggs inducing activity was measured. Each bar represents the mean ± SD of 6–10 animals. *P < 0.05; **P < 0.01. Asterisks indicate significant differences vs. PO injection of each strain. (**F**) Developmental timing of 100 larvae was observed in the *wt* and four mutants. The developmental period was considered the period when all larvae molted to the next instar (instars I–IV) and when all larvae initiated spinning (instar V). The number of parenthesis shows the number of larva died at this stage. **(G**–**H**) Eggs were incubated at 25 °C (25DD) or 15 °C (15DD) in the dark. Pupae (**G**) and cocoon-shell (**H**) weights after 4 d of pupation are shown in female. Each bar represents the mean ± SD of 35 animals. n.s., non-significant; *P < 0.05; ***P < 0.001. Orange asterisks indicate significant differences vs. *wt* (25DD); blue asterisks indicate significant differences vs. each 25DD strain. (Scale bar, 2 mm).

In general, diapause eggs have dark brown pigmentation because of 3-hydroxykynurenine (3-OHK) in their serosa, whereas non-diapause eggs lack this pigment, and thus, appear light yellow. Notably, DH was suggested to facilitate the accumulation of 3-OHK in pupal ovaries^14^. Progeny eggs of *wt* (25DD) showed light brown pigmentation on day 2 after oviposition, became dark brown on day 10 after oviposition, and, as they were diapause eggs, eventually failed to hatch (Fig. 3A, *wt*). Progeny eggs of Δ*DHP33* and Δ*DHR96* showed no pigmentation, and the larvae hatched on day 10 after oviposition despite 25DD rearing conditions (Fig. 3A, Δ*DHP33*, Δ*DHR96*). The percentage of diapause eggs was counted in 50 batches of progeny eggs among the *wt* and four mutants (Fig. 3C and Supplementary Fig. S1A). In the *wt*, 25DD and 20LL adults oviposited diapause eggs, and 15DD and 20DD eggs mostly became non-diapause eggs. However, all four mutant adults oviposited non-diapause eggs, mostly under 25DD, 15DD, 20LL, and 20DD conditions (Fig. 3C). Thus, disruption of the DH signaling pathway appeared to block diapause induction in the mutants despite diapause-inducing temperature and photoperiod conditions.

Interestingly, a few eggs in the mutants had light-brown pigmentation on day 2 after oviposition (Fig. 3B, black asterisk); these pigmented eggs hatched, but embryonic development was slightly delayed (Fig. 3B, white asterisk). Further, some of the pigmented eggs failed to hatch, and embryonic development was arrested at a specific stage during embryogenesis, immediately after formation of the cephalic lobe and telson and after segmentation of mesoderm, known as the diapausing stage in *wt* (Fig. 3D)^23^. The resulting mutants oviposited diapause eggs at ratios of 0.03–0.50% (Fig. 3C and Supplementary Fig. S2A). Next, we attempted rescue experiments of mutant lines by injecting synthetic DH or other FXPRLa to confirm whether only DH was responsible for diapause induction (Fig. 3E). In *wt* (15DD), DH had a significant effect on diapause egg inducing activity, which increased in a dose-dependent manner at the range of 10–1000 pmol/pupa; however, PBAN showed diapause egg inducting activity only at 100 times the amount of DH. Furthermore, almost no activity was observed after injections of α-, β-, and γ-SGNPs. In Δ*DHP33* and Δ*DHP531* lines, as well as in *wt* (15DD), high diapause eggs inducing activities were noted only after DH injection. In addition, almost no activity was observed in Δ*DHR96* and Δ*DHR111* even after DH injection.

Previous studies using *in vitro* assays showed that *Bombyx* DHR is also expressed in the prothoracic gland, the organ that synthesizes and releases the insect molting hormones (ecdysteroids), which may be activated by DH to function ecdysteroidogenesis in larval instars^8^. Therefore, we tested for effects on the developmental timing of ecdysis and metamorphosis in the mutant lines (Fig. 3F–H). Generally, *wt* (15DD) larvae spent less time in the larval period than did *wt* (25DD) larvae; *wt* (15DD) larvae initiated spinning earlier than did *wt* (25DD) larvae^23^, as indicated by the 3-day shift in *wt* (15DD) compared to that in *wt* (25DD) (Fig. 3F). We reared 100 larvae from each of the *wt* and four mutants, all under the same conditions, and observed the time of molting and duration of the larval period. Most *wt* (25DD) larvae spent 5, 4, 5, 2.5, and 7.5 d in the 1st, 2nd, 3rd, 4th, and 5th instar, respectively (Fig. 3F), and larvae initiated spinning 21 d after hatching, with a peak at 22 d (Supplementary Fig. S1B)—similar to that of the four mutants under 25DD conditions (Fig. 3F). Under 15DD conditions, the four mutants and *wt* showed similar developmental timing (Fig. 3F and Supplementary Fig. S1B). In addition, because it is known that *wt* (25DD) pupae have heavier bodies and cocoon shells^23^, we tested whether pupal and cocoon-shell weights are affected by DH signaling. Both female and male pupae incubated under 25DD conditions had bodies (Fig. 3G and Supplementary Fig. S1C) and cocoon shells (Fig. 3H and Supplementary Fig. S1D) that were heavier than those of *wt* (15DD) and that were similar in weight to those of all mutant pupae. Thus, we did not observe differences in the duration of the larval period or the weight of pupae and cocoons in *DH-PBAN* and *DHR* mutants, suggesting that DH signaling does not participate in ecdysteroidogenesis *in vivo*. Further, because the previous *in vitro* experiments used high concentrations of DH, we speculated that artificial effects were observed.

PBAN is known to stimulate the secretion of a sex pheromone, bombykol, from the pheromone gland in *Bombyx*^3^. Further, we observed a slight reduction in sexual behaviors such as flapping, orientation, and attempted copulation in male Δ*DHP33* and Δ*DHP531* mutants but not in Δ*DHR* mutants, which suggests that pheromone production is suppressed by PBAN knockout in female. Each mutant eventually mated and oviposited eggs in similar numbers to the *wt* (Supplementary Fig. S1A).

## Discussion

We clearly showed in this study that *in vivo* disruption of *DH-PBAN* and *DHR* blocked diapause induction in progeny embryos. As described previously, when expressed in a *Xenopus* oocyte system, DHR showed the highest affinity (EC_50_, ~70 nM) for DH compared with the other FXPRLa encoded by *DH-PBAN*^8^,^13^. Furthermore, an *in vivo* bioassay showed that synthetic DH was more effective than other FXPRLa at inducing diapause, with threshold levels less than 1/100 that of PBAN and other peptides encoded by *DH-PBAN* (Fig. 3E)^10^. In *Orgyia thyellina*, not only DH induced embryonic diapause in progeny, but also other FXPRLa encoded by *DH-PBAN* induced diapause in an *in vivo* bioassay^24^. Taken together, we conclude that DH signaling is essential for diapause induction and that a highly sensitive and specific interaction between DH and DHR is a result of ligand–receptor coevolution in *Bombyx mori*.

Extensive structural–functional studies of the *Bombyx* PBAN receptor (PBANR) have been performed using mutant receptors and *in vitro* assays^25^,^26^. These studies revealed a number of functional domains and sites that are crucial for receptor activation and regulation; thus, it has been suggested that the extracellular loops (regions between each transmembrane domain) of PBANR, DHR, and related GPCRs function as a ligand-selection filter^26^. Therefore, the extracellular loop domains of DHR may have evolved to interact selectively with DH as well as to fulfill the functional requirements for diapause induction in *Bombyx*. The Δ*DHR111* mutant, which was defective in eight amino acids of the extracellular N-terminus, had a similar phenotype as did the null mutant Δ*DHR96*. Since domain swaps in the *Helicoverpa zea* PBANR suggested roles for the N-terminus in ligand binding^5^, the Δ*DHR111* mutant may be defective in ligand binding ability. Furthermore, we showed that the DH titer increased in *DHR* mutants compared to the *wt*. It is probable that these mutant receptors were unable to internalize the ligand, similar to that reported for PBANR^27^, or were unable to trigger negative feedback regulation of DH release, resulting in an abnormal increase in DH titer. Thus, we propose, based on our TALEN-mediated *in vivo* analysis, that the extracellular N-terminus is critical for *Bombyx* DHR function.

The structural similarity between DH and CAPA-PK, which carries the WFGPRLa sequences in the C-terminus (Supplementary Table S1), has been assumed to explain the highly sensitive cross-reactivity of CAPA-PK to DHR. We clearly demonstrated the role of DH in diapause induction. It may be likely that there are differences in spatiotemporal dynamics between DH and CAPA-PK. Therefore, CAPA-PK might not interact with DHR in ovaries during pupal–adult development.

**Figure 4:**
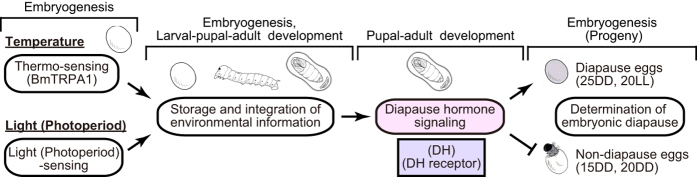
Schematic drawing of relationship between temperature and photoperiod and diapause induction via a unique peptidergic signaling system, DH signaling. Progeny diapause is determined by environmental temperature and photoperiod during maternal embryonic development. Silkworms incubated under 25DD and 20LL conditions lay pigmented diapause eggs. In contrast, incubation at 15DD and 20DD causes the resultant moths to lay non-diapause eggs. BmTRPA1 acts as a thermosensitive channel that affects diapause induction. We hypothesize that the links in the pathway from reception of environmental signals to expression of the diapause phenotype include storage, integration, and later translation of information into DH signaling in the form of diapause induction. Non-diapause eggs complete their embryogenesis approximately 9 d after oviposition at 25 °C. In contrast, diapause eggs remain in the diapause stage.

In facultative diapause, the decision to enter diapause is generally determined by environmental factors such as photoperiod, temperature, and nutrition received by that individual or its mother at an earlier developmental stage^28^. Although many links in the pathway leading from reception of environmental signals to expression of diapause phenotype remain poorly understood, it has been proposed that environmental information is stored, integrated, and later translated into neuroendocrine functions in the form of diapause induction^29^. The duration over which the information is stored may span numerous developmental stages or even generations^29^. We demonstrated that this hypothesis is well adapted to embryonic diapause in *Bombyx*. Recently, we showed that the embryonic BmTRPA1 acts as a thermosensitive channel that is activated at temperatures above ~21 °C and affects diapause induction through DH release during pupal–adult development^15^. In this study, we demonstrated that both thermal and photoperiod information was stored until the mid-pupal stage and was integrated with DH signaling to determine diapause phenotype, although the molecular mechanism(s) participating in light (photoperiod)-sensing and storage and integration of information remain unknown (Fig. 4). Furthermore, because it has been speculated that innervation from the brain controls the release of DH^30^, integrated information may affect brain plasticity in the control of DH release. Here, we have attempted to resolve the molecular mechanisms described in Fig. 4 using TALEN-based mutagenesis.

Diapause is accompanied by complex physiological and biochemical changes (referred to as diapause syndrome) in which reserves are accumulated prior to diapause to enable survival during diapause and post-diapause development^31^. In *Bombyx*, there are dramatic metabolic differences between 25DD and 15DD during the preparative phase of diapause in the maternal generation. Namely, 25DD eggs accumulate greater glycogen and become pigmented. As suggested in previous reports^14^,^21^,^22^, we revealed that DH signaling alone facilitates greater accumulation of glycogen as well as accumulation of 3-OHK in 25DD eggs. Because the removal of SG from diapause-type animals in mid-pupal stages can induce the production of non-diapause eggs that are occasionally light pink^32^, the mutant phenotypes of light-colored pigmented eggs obtained here are consistent with the idea that the animals were deficient in DH signaling.

Although the DH signaling cascade was blocked in the *DH-PBAN* and *DHR* mutants under 25DD, 20LL, and 20DD conditions, we obtained a few pigmented diapause eggs from these mutants. Largely unknown signaling pathway(s) may be active in the preparative phase of diapause induction during embryonic development at temperatures above 20 °C. For example, we observed extended developmental periods and heavier bodies and cocoon shells in 25DD silkworms, consistent with a previous report^23^. We suggest that the activation of BmTRPA1 signaling pathway(s) might affect the duration of growth in the larval period and the weight of the pupal body and cocoon shell. This signaling might be involved in preparing the diapause phenotype to facilitate entering diapause, and it might participate in the storage and integration of information linked to DH signaling. Therefore, 25DD conditions increase the potential for silkworms to enter diapause. Occasionally, diapause may be accidentally induced in a subset of eggs, without DH signaling. Although DH signaling is essential for diapause induction, unknown signaling pathway(s) including the BmTRPA1 pathway may help in preparing for diapause induction at 25 and 20 °C during embryogenesis. These pathways might also participate in the storage and integration of environmental information linked to DH signaling in the maternal generation in order to induce diapause.

## Materials and Methods

### Silkworms

The bivoltine (Kosetsu) strain of *Bombyx mori* was used in these experiments. Eggs were incubated under four different conditions: (1) at 25 °C under continuous darkness (25DD) to obtain diapause eggs in the wild type (*wt*); (2) at 15 °C under continuous darkness (15DD) to obtain non-diapause eggs in the *wt*; (3) at 20 °C under continuous illumination (20LL) to obtain diapause eggs in the *wt*; or (4) at 20 °C under continuous darkness (20DD) to obtain non-diapause eggs in the *wt*. Larvae were then reared on an artificial diet (Kuwano-hana, JA Zennoh Gunma) at 25–27 °C under a 13-h light/11-h dark cycle (13L:11D) and relative humidity of 30–50%. The percentages of diapause eggs were estimated by counting the numbers of eggs in diapause and those not in diapause after non-diapause eggs hatched in 50 egg batches. The results are expressed as the average percent diapause in each egg batch^14^.

To screen the knockout strain, eggs were incubated at 25 °C under high humidity (approximately 80%) until hatching; larvae were reared at 25 to 27 °C under long-day conditions (20L:4D) on an artificial diet (Product No. 404110, Kyoto Institute of Technology) to induce non-diapause eggs despite 25DD conditions, as described previously^33^.

### TALEN construction and screening of knockout silkworm

Construction of TALEN mRNAs and screening for germline mutants were performed according to Takasu *et al*.^17^. Briefly, TALEN targets were searched using TAL Effector Nucleotide Targeter 2.0 (https://tale-nt.cac.cornell.edu) in the coding regions of the *DH-PBAN* and *DHR* genes. DNA constructs containing the TAL segments were prepared using Golden Gate TALEN kit (Addgene). TALEN mRNAs were then synthesized using mMessage mMachine T7 Ultra kit (Ambion); mRNA of each TALEN was mixed at a concentration of 0.5 μg/μL for microinjection. Non-diapause eggs of the Kosetsu strain were collected within 1 h after oviposition during the syncytial blastoderm stage; the TALEN mRNA mixture was injected into the eggs using a glass needle (uMPm-02; Daiwa Union) attached to a manipulator (kaikopuchu-STDU1; Daiwa Union) and FemtoJet (Eppendorf).

For screening of germline mutagenesis, the G_0_ adults were mated with *wt*. The oviposited G_1_ eggs were collected, and approximately 10 eggs from each brood were pooled for genomic DNA extraction using Nucleospin Tissue (Macherey-Nagel). The DNA fragment containing the targeted region of interest was amplified by PCR using Takara Ex Taq (Takara) (Supplementary Fig. S2A). To test for mutagenesis, the PCR products of *DH-PBAN* and *DHR* were digested with restriction enzymes *Psp*1406I (Takara) and FastDigest *Mnl*I (Thermo), respectively; the presence of an undigested PCR product would suggest that the restriction site was disrupted by TALENs (Supplementary Fig. S2A,B). Mutated PCR products were subcloned using a TOPO TA cloning kit (Invitrogen) and checked by sequencing. The broods containing mutated sequences were reared, and mutated G_1_ adults were crossed with the siblings that carried the same mutation. Homozygous mutants were obtained after confirmation by sequencing of the target region in the G_2_ or G_3_ egg genome.

### Immunostaining

The immunostaining procedures were adapted from Hagino *et al*.^20^. Briefly, the primary antibodies, anti–DH[N] or –PBAN[N], which recognize a 12–amino acid sequence of the N–terminal region of each peptide, were used at a ratio of 1:2500, respectively, at 4 °C overnight. The signal was detected with Cy2–labeled goat anti–mouse IgG (Jackson ImmunoResearch Lab.) diluted to 1:1500 and was observed using an Olympus FV1000–D confocal microscope (Olympus). Confocal scans were performed under the same conditions for specimens in each mutant strain.

### Time-resolved fluoroimmunoassay (TR-FIA)

We developed a new method for measurement of the hemolymph DH titers. Hemolymph was collected on ice from one or two pupae on day 4 after pupation into a microcentrifuge tube containing small amounts of sodium diethyldithiocarbamate, a phenoloxidase inhibitor, and *p*-APMSF, a protease inhibitor. The final concentrations of the inhibitors were 5 mM and 20 μM, respectively. After centrifuging at 9,200 × *g* for 5 min to remove hemocytes, 150 μL of the hemolymph was added with 150 μL of 2% acetic acid and 300 μL of methanol, followed by boiling for 10 min. The mixture was centrifuged at 18,000 × *g* and 480 μl of the resulting supernatant was concentrated to approximately 100 μl by vacuum centrifugation for 1 h, followed by mixing with 1 ml of 1% trifluoroacetic acid (TFA). This mixture was applied to a Sep-Pak Vac 3cc C8 cartridge (Waters) equilibrated with 0.1% TFA. The cartridge was washed with 10% acetonitrile (ACN) containing 0.1% TFA and eluted with 40% ACN containing 0.1% TFA. The eluate was lyophilized and then dissolved with dilution buffer [TBS (50 mM Tris-HCl containing 0.9% NaCl) containing 0.5% BSA, 0.1% Tween-20, and 0.05% sodium azide] for use in DH determination by TR-FIA. The recovery rate of DH by this extraction method was estimated to be ≈80%.

TR-FIA was developed based on the method described by Mizoguchi *et al*.^34^. The wells of an RIA/EIA plate (Costar #3590) were filled with 80 μL each of anti-DH[N] mouse monoclonal antibody^35^ (1.5 μg/mL in TBS) and incubated overnight at 4 °C. After discarding the antibody solution, the wells were blocked with TBS containing 4% skimmed milk and 0.1% Tween-20 for 1 h at 25 °C. After washing three times with TBS-T (TBS containing 0.05% Tween-20), 100 μL of anti-DH[C] rabbit antibody^35^ diluted 1:300 with dilution buffer and either the standard hormone (chemically synthesized DH in 50 μL dilution buffer) or 50 μL of the test sample (hemolymph extract) were distributed into the wells, and the plate was incubated for 2 h at 25 °C with shaking on a microplate shaker. The wells were then washed four times with TBS–T, filled with 50 μL of DELFIA Assay buffer (PerkinElmer) containing biotinylated anti–rabbit IgG antibody (Boehringer Mannheim) and europium–labeled streptavidin (PerkinElmer) at concentrations of 83 and 200 ng/mL, respectively, and incubated for 1 h at 25 °C with shaking. After incubation, the wells were developed with DELFIA Enhancement solution and the fluorescence signals measured with an ARVO X4 plate reader (PerkinElmer).

### Measurement of glycogen content

The ovaries were dissected out with phosphate-buffered saline (PBS) just after eclosion. One ovariole was thoroughly separated from each animal, blotted dry, weighed quickly, and stored at –20 °C before use. Glycogen was extracted by digesting the homogenate with 30% (w/v) KOH in a boiling bath for 30 min; the glycogen was then precipitated with ethanol at 4 °C^36^ and measured by the phenol-sulfuric acid method^37^.

### Thionin staining of embryo

The embryos were collected 10 d after oviposition and were stained with carbolic thionin solution according to An *et al*.^38^ with modifications; to facilitate dechorionization, the eggs were boiled in 80% ethanol for 5 min after fixation.

### Rescue experiment

Synthetic peptides (DH, α-, β-, and γ-SGNPs, and PBAN) of 95% purity (HPLC area percentage) were obtained from Operon Biotechnologies. Each peptide was dissolved in peanut oil (Sigma-Aldrich), and 10 μl solutions of various doses were injected into pupa at 4 days after pupation. The diapause eggs inducing activity was estimated by counting the numbers of eggs in diapause and those not in diapause after the non-diapause eggs hatched. The results are expressed as the average percent diapause in each egg batch as described above.

### Statistical analysis

Data were compared using Student’s *t*-tests. The significance of differences presented in Fig. 3E was evaluated using the Steel–Dwass test. Statistical analyses were performed in Excel 2011 (Microsoft) with the software add-in Toukei-Kaiseki Ver. 2.0 (Esumi).

## Additional Information

**How to cite this article**: Shiomi, K. *et al*. Disruption of diapause induction by TALEN-based gene mutagenesis in relation to a unique neuropeptide signaling pathway in *Bombyx*. *Sci. Rep*. **5**, 15566; doi: 10.1038/srep15566 (2015).

## Supplementary Material

Supplementary Information
