# Supplementary material for: Disruption of diapause induction by TALEN-based gene mutagenesis in relation to a unique neuropeptide signaling pathway in Bombyx
Source: Sci Rep. 2015 Oct 26;5:15566. doi: 10.1038/srep15566 (PMC4620438; doi:10.1038/srep15566)
Supplement: Supplementary Information [file srep15566-s1.pdf]

**(Supplementary Information)**

Disruption of diapause induction by TALEN-based gene mutagenesis in relation to a unique neuropeptide signaling pathway in *Bombyx*

Kunihiro Shiomi[1‡], Yoko Takasu[2], Masayo Kunii[1], Ryoma Tsuchiya[1], Moeka Mukaida[1], Masakazu Kobayashi[1], Hideki Sezutsu[2], Masatoshi Ichida (Takahama)[3], Akira Mizoguchi[4]

[1]Faculty of Textile Science and Technology, Shinshu University, Ueda 386-8567, Japan, [2]National Institute of Agrobiological Sciences (NIAS), Tsukuba 305-8602, Japan, [3]Center for Bioresource Field Science (CBFS), Kyoto Institute of Technology, Kyoto 606-8585, Japan, [4]Graduate School of Science, Nagoya University, Nagoya 464-8602, Japan

Peptides predicted based on the *capa* and *DH-PBAN* gene sequences in *Bombyx mori*

| Gene | (Accession No.) | Peptide | Sequence | Receptor (Accession No.) |
|---|---|---|---|---|
| *capa* | (NP_001124357.1) | CAPA-PVK1 | PDGVLNLYPFPRVa | N.A. * |
| | | CAPA-PVK2 | QLYAFPRVa | N.A. * |
| | | CAPA-PK | NEPHDDLLGLHLDDPGMWFGPRLa | N.A. |
| *DH-PBAN* | (NP_001037321.1) | DH | TDMKDESDRGAHSERGALWFGPRLa | AB164386 |
| | | α-SGNP | IIFTPKLa | ** |
| | | β-SGNP | SVAKPQTHESLEFIPRLa | ** |
| | | PBAN | LSEDMPATPADQEMYQPDPEEMESRTRYFSPRLa | AB181298 |
| | | γ-SGNP | TMSFSPRLa | ** |

(ref. 3, 9, 11)
N.A. indicates no analysis of ligand-receptor interaction by in vitro assay.
* indicates that orthologs of capa receptor in *Drosophila melanogaster* were described as BNGR-A25, BNGR-A27 in (11)
** indicates that those ligands and DH or PBAN receptor interactions were analyzed in (8,13).

Supplementary Table S1. (Shiomi et al. 2015)

A

| | | (The number of diapause eggs in relation to total numbers of eggs) | | | |
|---|---|---|---|---|---|
| | *N* (egg batch) | 25DD | 15DD | 20LL | 20DD |
| *wt* | 50 | (9,910 / 9,910) | (0 / 6,670) | (10,904 / 10,904) | (181 / 10,055) |
| Δ*DHP33* | 50 | (19 / 8,865) | (0 / 6,889) | (14 / 9,332) | (9 / 9,393) |
| Δ*DHP531* | 50 | (49 / 9,274) | (0 / 7,110) | (5 / 10,751) | (6 / 9,986) |
| Δ*DHR96* | 50 | (17 / 10,529) | (0 / 5,278) | (4 / 9,987) | (5 / 10,429) |
| Δ*DHR111* | 50 | (7 / 9,802) | (0 / 7,123) | (2 / 8,508) | (0 / 8,056) |

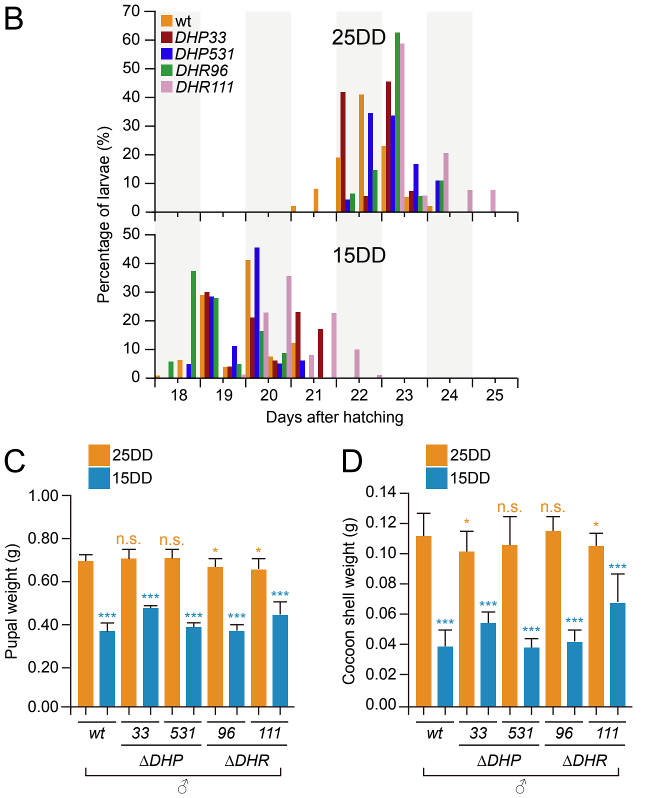


**Supplementary Figure S1.** (**A**) The number of diapause eggs in relation to total numbers of eggs. These data are based on Fig. 3C. (**B**) Initiation of spinning in 100 larvae each of *wt* and four mutants. The percentage of larvae is represented as days between hatching and initiation of spinning in final- (fifth-) instar larvae. The larvae were checked twice daily, at 8:00 AM and 5:00 PM. (**C**, **D**) Effects of mutations on pupal and cocoon-shell weights in male. Eggs were incubated at 25 ° C (25DD) or 15 ° C (15DD) in the dark. Pupae (**C**) and cocoon-shell (**D**) weights after 4 d of pupation are shown. Each strain used 35 animals ($N$ = 35). Data are means ± SD. n.s., non-significant; *, $P < 0.05$; ***, $P < 0.001$. Orange asterisks indicate significant differences vs. *wt* (25DD); blue asterisks indicate significant differences vs. each 25DD strain.

Supplementary Fig. S1. (Shiomi et al. 2015)

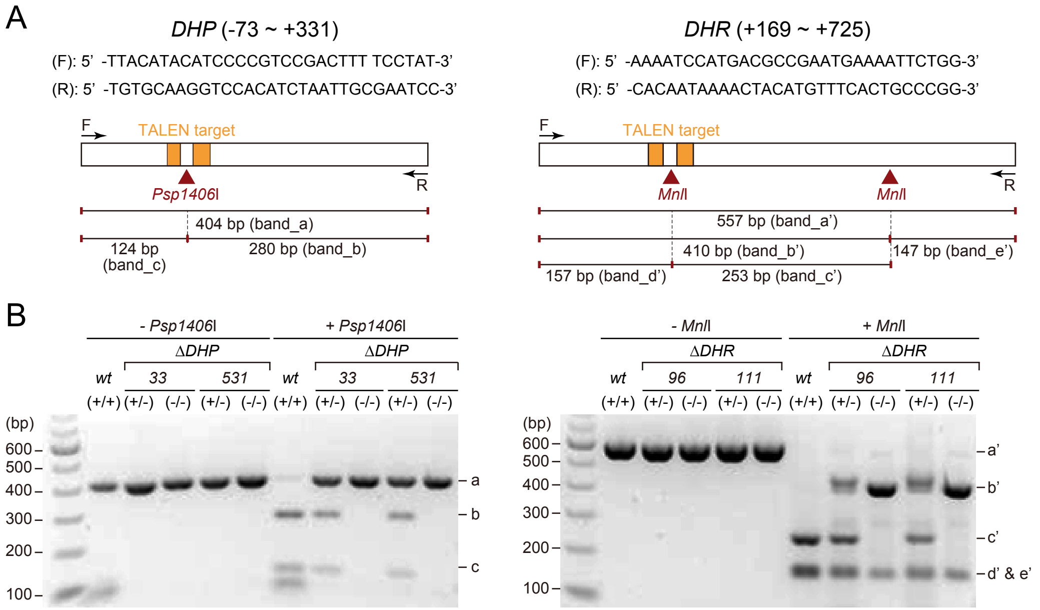


**Supplementary Figure S2.** Screening of the mutant lines. (**A**) The genomic DNA was extracted from eggs of each brood. Each DNA fragment of *DH-PBAN* (-73 to +331) and *DHR* (+169 to +725) containing the targeted region of TALEN was amplified by PCR using primers F and R. To check for mutagenesis, the PCR products of *DH-PBAN* and *DHR* were digested with restriction enzymes *Psp1406*I and *Mnl*I, respectively; the presence of an undigested PCR product would suggest that the restriction site was disrupted by TALENs. (**B**) Gel images of restriction enzyme digested PCR products from *wt* and four mutant lines (Δ*DHP33*, Δ*DHP531*, Δ*DHR96*, and Δ*DHR111*) digested with or without enzymes. The genomic DNAs from heterozygous (+/-) and homozygous (-/-) mutants were used in PCR, and further subjected to restriction enzyme digestion. The bands (a–c, a′–e′) corresponded to those in Figure S2A.

Supplementary Fig. S2. (Shiomi et al. 2015)
